# Effect of Surface Modifications on Surface Roughness of Ti6Al4V Alloy Manufactured by 3D Printing, Casting, and Wrought

**DOI:** 10.3390/ma16113989

**Published:** 2023-05-26

**Authors:** János Kónya, Hajnalka Hargitai, Hassanen Jaber, Péter Pinke, Tünde Anna Kovács

**Affiliations:** 1 Doctoral School on Materials Sciences and Technologies, Óbuda University, Bécsi út. 96/B., H-1034 Budapest, Hungary; janos@dentarttechnik.hu; 2 Dent-Art Technik Ltd., Csokonai u. 10., H-9024 Győr, Hungary; 3 Department of Materials Science and Technology, Széchenyi István University, Egyetem tér 1., H-9026 Győr, Hungary; hargitai@sze.hu; 4 Bánki Donát Faculty of Mechanical and Safety Engineering, Óbuda University, Népszínház u. 8., H-1081 Budapest, Hungary; hassen.jaber@bgk.uni-obuda.hu (H.J.); pinke.peter@bgk.uni-obuda.hu (P.P.)

**Keywords:** surface roughness, 3D printing, Ti6Al4V, selective laser melting (SLM)

## Abstract

This work aimed to comprehensively evaluate the influence of different surface modifications on the surface roughness of Ti6Al4V alloys produced by selective laser melting (SLM), casting and wrought. The Ti6Al4V surface was treated using blasting with Al_2_O_3_ (70–100 µm) and ZrO_2_ (50–130 µm) particles, acid etching with 0.017 mol/dm^3^ hydrofluoric acids (HF) for 120 s, and a combination of blasting and acid etching (SLA). It was found that the optimization of the surface roughness of Ti6Al4V parts produced by SLM differs significantly from those produced by casting or wrought processes. Experimental results showed that Ti6Al4V alloys produced by SLM and blasting with Al_2_O_3_ followed by HF etching had a higher surface roughness (Ra = 2.043 µm, Rz = 11.742 µm), whereas cast and wrought Ti6Al4V components had surface roughness values of (Ra = 1.466, Rz = 9.428 m) and (Ra = 0.940, Rz = 7.963 m), respectively. For Ti6Al4V parts blasted with ZrO_2_ and then etched by HF, the wrought Ti6Al4V parts exhibited higher surface roughness (Ra = 1.631 µm, Rz = 10.953 µm) than the SLM Ti6Al4V parts (Ra = 1.336 µm, Rz = 10.353 µm) and the cast Ti6Al4V parts (Ra = 1.075 µm, Rz = 8.904 µm).

## 1. Introduction

Osseointegration is a crucial factor in the success of dental and bone implants [1]. The term is generally understood to mean the formation of good interaction and functional connection between the surface of an implant and living bone tissue. As a result, the osseointegration process is strongly influenced by implant surface conditions such as surface roughness, chemical composition, charge, and energy [2]. The surface roughness is recognized as being the most important parameter influencing the speed and quality of osseointegration [3]. There are three categories of surface roughness based on size: macro-rough (10–30 µm), micro-rough (1–10 µm), and nano-rough (less than 1 µm). It is shown that an increase in the macro-, micro-, and nano-structured surface morphologies can improve osseointegration and enhance bone fixation [4,5]. Therefore, dental implant quality is directly dependent on surface conditions. To improve the osseointegration of dental implants, surface modification technologies are often used, such as blasting, polishing, chemicals (acid etching), and blasting plus acid etching (SLA) [6]. In blasting, hard ceramic particles are shot through a nozzle into the surface of implants utilizing compressed air at high speed. Acid etching treatment involves immersing the implants in a strong acid such as hydrofluoric acid (HF), nitric acid (HNO_3_), and/or sulphuric acid (H_2_SO_4_). The SLA is blasting followed by acid etching. Souza et al. [6] investigated the effect of blasting followed by acid etching (SLA) on the proteomic profile of layers of proteins adsorbed from saliva and blood plasma on the surface of a Ti-Zr alloy. Wang et al. [7] studied the impact of the processing parameters of electron beam melting (EBM) on the surface roughness of manufactured parts. Szymczyk-Zi’ołkowska et al. [8] investigated the influence of surface modifications (polishing, sandblasting, and acid-polishing) of Ti6Al4V implants produced by EBM on essential biological properties (wettability, cytotoxicity, and biofilm formation). They concluded that surface modification has a very strong influence on biological properties.

Titanium alloys, especially Ti6Al4V, are an important alloy for dental and orthopaedic implants owing to their excellent mechanical and biological properties [9]. In recent years, there has been increased interest in the use of 3D printing technology (selective laser melting, SLM) for the manufacture of Ti64 medical implants in place of powder metallurgy, wrought and casting processes [10,11]. In this work, the surface roughness in terms of arithmetic mean roughness (Ra) and mean depth of roughness (Rz) of Ti6Al4V samples manufactured by SLM, casting, and wrought were measured and compared. It was found that the surface roughness was different for each process.

## 2. Materials and Methods

### 2.1. Preparation of Ti6Al4V Samples

**Figure 1:**
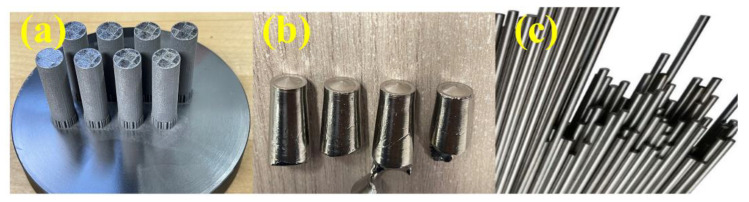
(**a**) 3D-printed, (**b**) Casting, and (**c**) wrought specimens.

**Figure 2:**
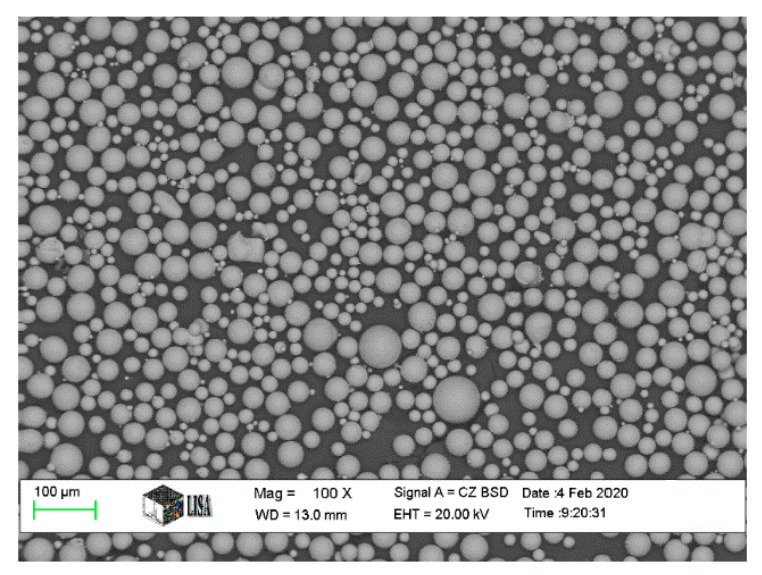
SEM micrograph shows the morphology of Ti6Al4V powder.

Polished cylindrical specimens of Ti6Al4V produced by three methods, SLM, casting and wrought, were used as the base material to study the surface roughness (Figure 1). The SLM Ti6Al4V specimens (Figure 1a) were fabricated using an SLM machine (Sisma MYSINT 100, Via dell’Industria, Vicenza, Italia) equipped with a 200 W fibre laser and a 55 µm laser spot. The dimensions of the SLM Ti6Al4V samples were 9 mm in diameter, and 50 mm in height. The optimal settings consisted of a continuous laser power of 125 W, a scanning speed of 1000 mm/s, and a layer thickness of 20 µm. A constant flow of 35 L of argon gas per minute was used for protection. The starting material for SLM Ti6Al4V specimens was Ti6Al4V plasma-atomized spherical powder (Gr.5) provided by LPW Technology (Runcorn UK), as shown in Figure 2. The chemical composition of Ti64 powder is shown in Table 1. The size distribution ranged from 15 to 45 µm.

The casting Ti6Al4V specimens (Figure 1b) were fabricated using a vacuum-pressure, plasma jet-heated casting machine. To ensure better chemical homogeneity, the 20 g ingots were remelted three times. A red copper mould was used to cast the experimental alloys used in this investigation. The mould was a truncated cone which had a 10 mm top diameter and 14 mm base diameter and a height of 50 mm (Figure 1b). In addition, Ti6Al4V drawn-rolled specimens (Figure 1c), in its wrought condition, were used as the base metal, and was 9 mm in diameter. After the manufacturing process, all samples were subjected to a polishing process. The polishing process was performed by a WP-EX 2000 machine (Wassermann, Hamburg, Germany) equipped with rag polishing discs. The samples were polished with a #1200 grit SiC foil.

### 2.2. Surface Modification Technologies

The manufactured and polished samples were divided into three groups: Casting, wrought, and 3D Printing. Each group was subjected to five types of surface modification (see below).

1. Etched in 0.017 mol/dm^3^ of hydrofluoric acid (HF) for 120 s at room temperature.
2. Blasted with Al_2_O_3_ particles (70–100 μm) with 4 bar blasting pressure. The blasting was performed with a Renfert Basic Quattro IS.
3. Blasted with Al_2_O_3_ particles and etched in 0.017 mol/dm^3^ of hydrofluoric acid (HF) for 120 s at room temperature.
4. Blasted with ZrO_2_ particles (50–130 μm) with 4 bar blasting pressure.
5. Blasted with ZrO_2_ and etched in 0.017 mol/dm^3^ of hydrofluoric acid (HF) for 120 s at room temperature.

### 2.3. Surface Roughness and Topography

The Ra and Rz surface roughness were determined using an ALICONA Infinite Focus equipped with Vision software. For each surface, five measurements were performed.

## 3. Results and Discussion

The values of surface roughness, Ra and Rz, for all specimens, are detailed in Table 2. It is revealed that 3D-printed (SLM) Ti6Al4V components are significantly different from cast and wrought Ti6Al4V parts when it comes to optimizing surface roughness by surface treatments such as Al_2_O_3_ blasting + HF etching. Ti6Al4V alloys produced by 3D printing and blasting with Al_2_O_3_ followed by HF etching exhibit the highest surface roughness compared to cast and wrought Ti6Al4V parts. The surface roughness of the 3D-printed samples is the roughest (Ra = 2.043, Rz = 11.742 µm), followed by the surface of the cast samples (Ra = 1.466, Rz = 9.428 µm), and the surface of the wrought samples (Ra = 0.940, Rz = 7.963 µm). The increase in surface roughness and the change in surface morphology of Ti alloys have been reported in sandblasting and acid etching processes [13].

It is interesting to note that the surface treatment (ZrO_2_ blasting + HF etching) of the wrought Ti6Al4V parts has a higher surface roughness (Ra = 1.631, Rz = 10.953 µm) than the cast parts (Ra = 1.075, Rz = 8.904 µm) and the 3D-printed ones (Ra = 1.336, Rz = 10.353 µm). The reason for this is probably the difference in the surface properties of the manufactured samples, which leads to different inclusion of ejected particles on the surface of the samples. In addition, as can be seen from Table 2, the HF etching process leads to a reduction in the surface roughness of the polished cast specimen (polishing and then etching) from (Ra = 0.503, Rz = 3.573 µm) to (Ra = 0.344, Rz = 2.723 µm), as the surface oxidation removes material, resulting in the ionization of atoms. In the wrought sample (polishing then etching), the roughness remains the same without an increase or decrease. On the other hand, the HF etching process leads to an increase in the surface roughness of the polished 3D sample (polishing and then etching). This is due to the high hardness of the 3D-printed sample, which reduces the oxidation process. It has been reported that the hardness of specimens manufactured by 3D printing (SLM) (377 HV) [10] is higher than those manufactured by casting (340 HV) [14] or wrought (306 HV) [15].

### 3.1. Casting

**Figure 3:**
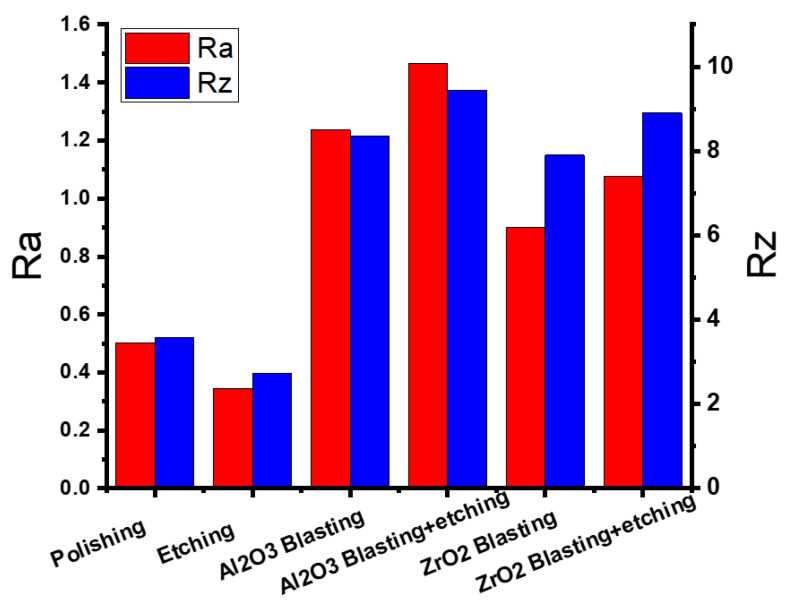
Results of the surface roughness of the cast-polished and surface-modified specimens.

**Figure 4:**
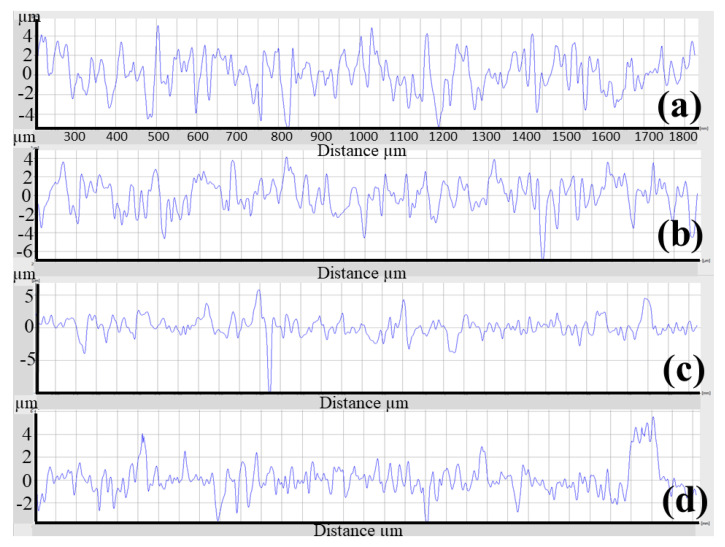
Surface roughness profile for each condition of the cast specimen. (**a**) Blasting with Al_2_O_3_ and etching with HF, (**b**) blasting with Al_2_O_3_, (**c**) blasting with ZrO_2_ and etching with HF, and (**d**) blasting with ZrO_2_.

**Figure 5:**
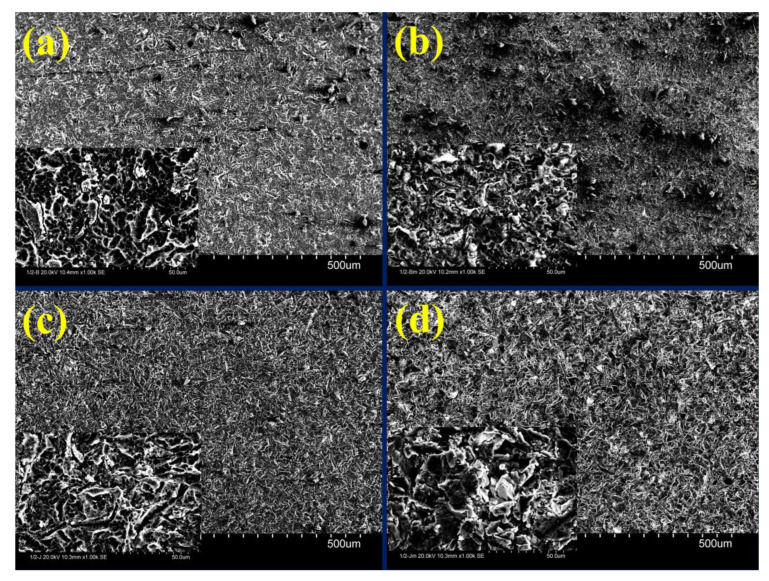
SEM micrographs for each condition of the cast specimen. (**a**) Blasting with ZrO_2_, (**b**) blasting with ZrO_2_ and etching with HF, (**c**) blasting with Al_2_O_3_, and (**d**) blasting with Al_2_O_3_ and etching with HF.

Figure 3 compares the surface roughness of the as-polished casted samples with samples after etching, blasting with Al_2_O_3_ or ZrO_2_, or a combination of these methods. As can be seen, the surface roughness was reduced from (Ra = 0.503, Rz = 3.573 µm) to (Ra = 0.344, Rz = 2.723 µm) by etching, and increased to (Ra = 1.236, Rz = 8.359 µm) by blasting with Al_2_O_3_, (Ra = 1.466, Rz = 9.428 µm) by blasting with Al_2_O_3_ and etching, (Ra = 0.900, Rz = 7.898 µm) by blasting with ZrO_2_, and (Ra = 1.075, Rz = 8.904 µm) by blasting with ZrO_2_ and etching. The highest surface roughness was achieved after a combination of blasting with Al_2_O_3_ and etching. This is confirmed by the surface roughness profile (Figure 4a) of the sample after Al_2_O_3_ blasting and etching. The surface is rougher than that of the other samples (Figure 4b–d). In addition, Figure 4a shows the alternation of sharp peaks with a height of 4 μm and sharp valleys with a depth of 6 μm. Figure 5 shows the surfaces of the samples when (a) blasting with ZrO_2_, (b) blasting with ZrO_2_ and etching with HF, (c) blasting with Al_2_O_3_, and (d) blasting with Al_2_O_3_ and etching with HF. In the blasting with ZrO_2_ (Figure 5a) and blasting with Al_2_O_3_ (Figure 5c) conditions, it can be seen that the sandblasted surface displayed an anisotropic structure of craters, valleys and peaks due to plastic deformation caused by the impact of Al_2_O_3_ and ZrO_2_ particles, and there may well be some particles embedded in the surface. During the plastic deformation process some materials can be removed from the surface [16]. The SEM images of the surface blasted with Al_2_O_3_ (Figure 5c) are identical to the images of the surface blasted with ZrO_2_ (Figure 5a). It is also possible to see the disordered position of the valleys and peaks produced. Figure 5b and d show the surfaces of the sample blasted with ZrO_2_ and etched with HF and the sample blasted with Al_2_O_3_ and etched with HF. As can be seen, the etching process produced a very rough surface due to surface cleaning as well as material removal from the surface due to oxidation. The preferential dissolution of the alpha phase of the Ti6Al4V alloys has been reported in an etching by HF [13]. After HF etching, the Al_2_O_3_-blasted surface becomes sharper in appearance (Figure 4a). Rounded peaks (Figure 4b) become sharp, which is confirmed by the surface roughness profile (Figure 4a).

### 3.2. Wrought

**Figure 6:**
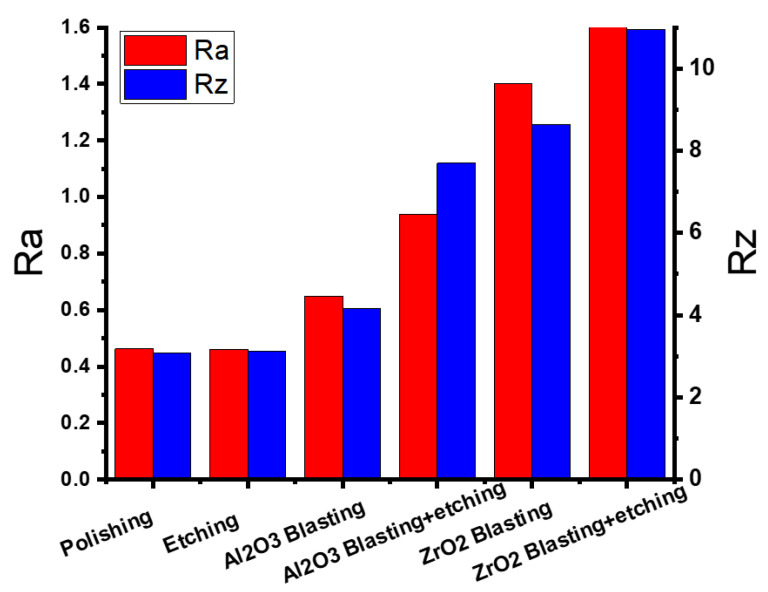
Results of the surface roughness of the wrought-polished and surface-modified specimens.

**Figure 7:**
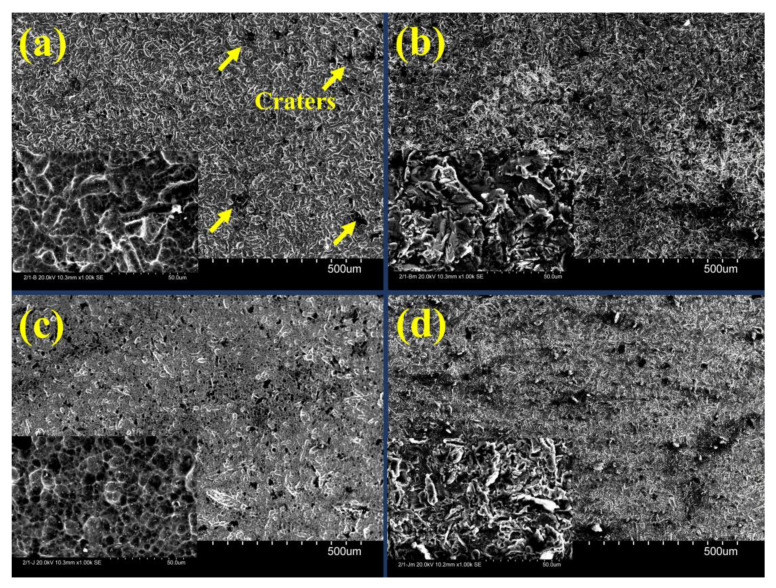
SEM micrographs for each condition of the wrought specimen. (**a**) Blasting with ZrO_2_, (**b**) blasting with ZrO_2_ and etching with HF, (**c**) blasting with Al_2_O_3_, and (**d**) blasting with Al_2_O_3_ and etching with HF.

**Figure 8:**
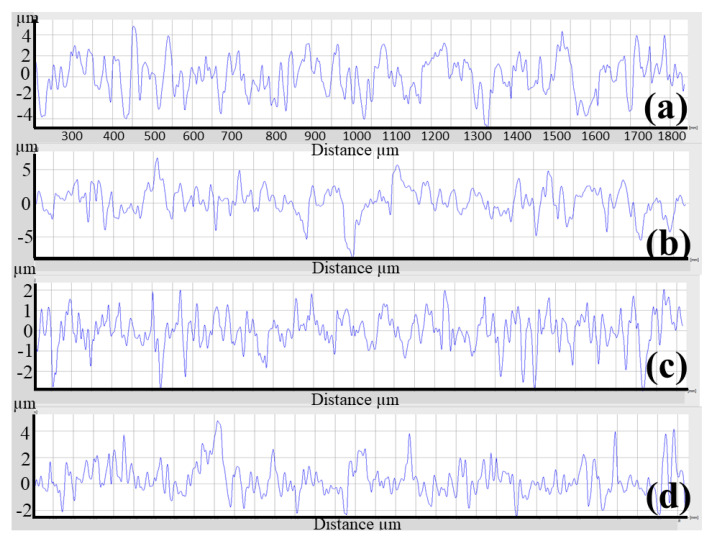
Surface roughness profile for each condition of the wrought specimen. (**a**) Blasting with ZrO_2_, (**b**) blasting with ZrO_2_ and etching with HF, (**c**) blasting with Al_2_O_3_, and (**d**) blasting with Al_2_O_3_ and etching with HF.

Figure 6 shows the surface roughness of the as-polished wrought manufactured Ti6Al4V components after various surface modifications. As can be seen, there is a remarkable increase in surface roughness from (Ra = 0.463, Rz = 3.086 µm) (in the as-polished condition) to (Ra = 0.650, Rz = 4.171 µm) after blasting with Al_2_O_3_, (Ra = 0.940, Rz = 7.693 µm) after blasting with Al_2_O_3_ and etching, (Ra = 1.401, Rz = 8.644 µm) after blasting with ZrO_2_, and (Ra = 1.631, Rz = 10.953 µm) after blasting with ZrO_2_ and etching. It should be noted that the surface roughness of the etched sample (Ra = 0.462, Rz = 3.122 µm) is the same as in the polished state (Ra = 0.463, Rz = 3.086 µm), without any change. The surface of Ti6Al4V after ZrO_2_ blasting was characterized by the presence of several craters, as shown in Figure 7a. The formation of craters could be attributed to ZrO_2_ abrasive particles. After ZrO_2_ blasting and etching (Figure 7b), a change in the surface was noticeable. Etching cleans the surface and removes material, resulting in a very rough surface. Figure 8 shows the roughness profile of each condition. After ZrO_2_ blasting and etching (Figure 8b), the surface shows several peaks (6 μm)-to-valley (6 μm) relationships, indicating that the surface became rougher after acid etching (Ra = 1.631, Rz = 10.953 µm) compared to the blasted ZrO_2_ sample (Ra = 1.401, Rz = 8.644 µm) and the other conditions. Figure 7c,d consists of SEM micrographs of the blasting with Al_2_O_3_ and blasting with Al_2_O_3_ and etching, indicating some small valleys and peaks, which are confirmed by Figure 8c,d to be of less roughness than the sample after ZrO_2_ blasting and etching. The surface blasted with Al_2_O_3_ exhibited regular and homogeneous pore features. After blasting with Al_2_O_3_, more uniform and smaller micro-rough valleys (average 7 μm in diameter) formed on the surface than in the other conditions. Similar surface characteristics were observed in previous results [17]. A distinct surface change can be observed on the rolled specimen after Al_2_O_3_ blasting. The surface topography consists of valleys (3 μm) and peaks (2 μm), as shown in Figure 8c. In addition, the peaks and valleys are present in approximately equal proportions.

### 3.3. 3D Printing

**Figure 9:**
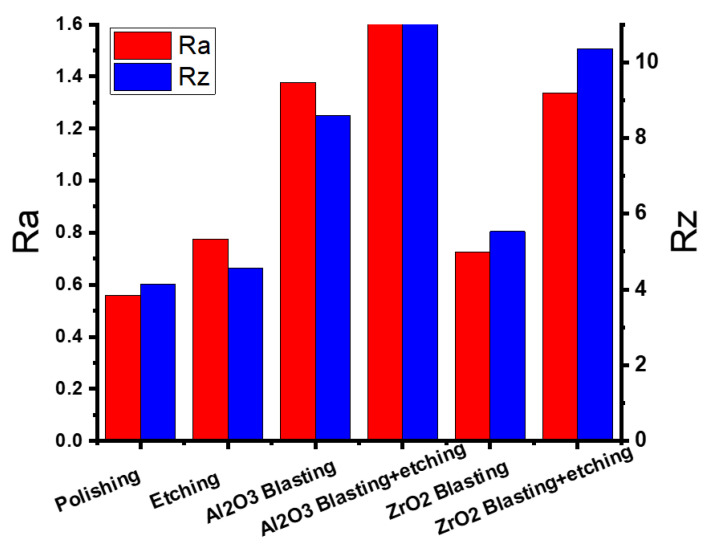
Results of the surface roughness of the 3D-polished and surface-modified specimens.

**Figure 10:**
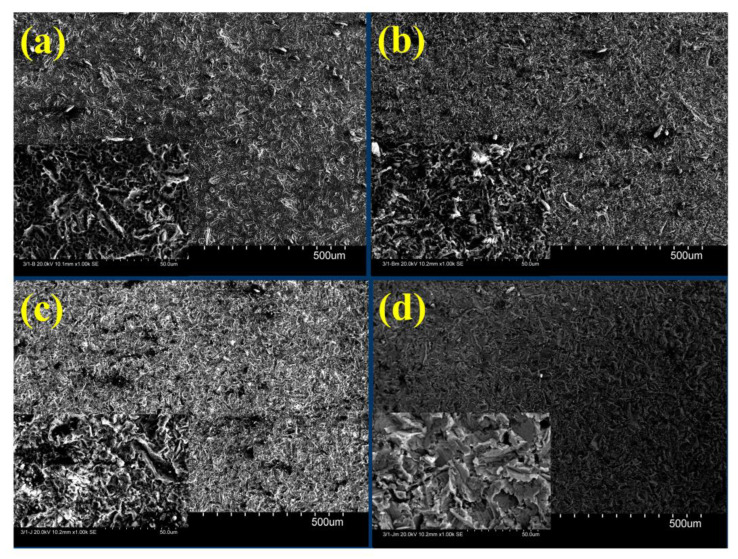
SEM micrographs for each condition of the 3D specimen. (**a**) Blasting with ZrO_2_, (**b**) blasting with ZrO_2_ and etching with HF, (**c**) blasting with Al_2_O_3_, and (**d**) blasting with Al_2_O_3_ and etching with HF.

**Figure 11:**
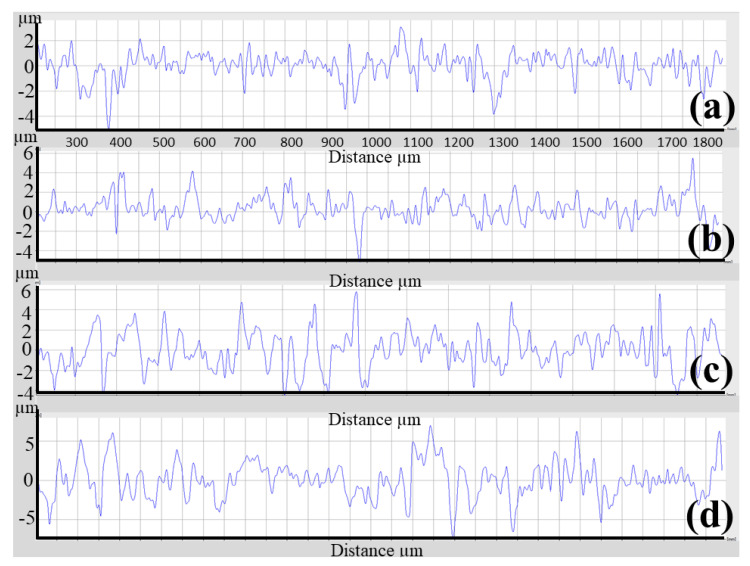
Surface roughness profile for each condition of the SLM specimen. (**a**) Blasting with ZrO_2_, (**b**) blasting with ZrO_2_ and etching with HF, (**c**) blasting with Al_2_O_3_, and (**d**) blasting with Al_2_O_3_ and etching with HF.

The as-polished 3D-printed sample had Ra and Rz values of 0.559 and 4.149 μm, respectively. Etching with HF, blasting with Al_2_O_3_, blasting with Al_2_O_3_ and etching, blasting with ZrO_2_, and blasting with ZrO_2_ and etching, increased Ra and Rz to 0.776 and 4.561 μm, 1.377 and 8.594 μm, 2.043 and 11.742 μm, 0.726 and 5.533 μm, and 1.336 and 10.353 μm, respectively, as shown in Figure 9. Figure 10a–d highlights the corresponding SEM micrographs after blasting with ZrO_2_, blasting with ZrO_2_ and etching, blasting with Al_2_O_3_, and blasting with Al_2_O_3_ and etching. It was noted that Figure 10d appears rougher because there are more valleys and cavities on the surface. Figure 11 shows the roughness profile difference between each condition. The absolute difference between the roughness profile of blasting with Al_2_O_3_ and etching (Figure 11d) and other samples (Figure 11a–c) was the surface topography, consisting of deep valleys (6 μm) and sharp peaks (6 μm). These results show that the surface modification process (blasting with Al_2_O_3_ and etching) is a suitable process to obtain the highest surface roughness of the produced titanium alloys. Furthermore, this surface roughness is described as a hierarchical structure composed of three different types of surface roughness based on dimensions: macro-rough (10–30 µm), micro-rough (1–10 µm), and nano-rough (less than 1 µm), all of which are advantageous to the osseointegration process [18].

## 4. Conclusions

The effects of surface modifications on the surface roughness of Ti6Al4V alloy components produced by 3D printing, casting, and wrought have been studied in detail. The following conclusions can be drawn from the results.

1. Significant differences were found in the surface roughness of specimens produced by 3D printing compared to those produced by casting and wrought after surface modifications were performed. This can be attributed to the difference in the surface properties of the manufactured samples, which leads to different inclusion of ejected particles on the surface of the samples.
2. The highest outcomes were collected for Ti6Al4V alloys fabricated using SLM and blasting with Al_2_O_3_, followed by HF etching (Ra = 2.043, Rz = 11.742 µm), or with Ti6Al4V fabricated using wrought and blasting with ZrO_2_, followed by HF etching (Ra = 1.631, Rz = 10.953 µm).
3. The surface roughness of the SLM-fabricated samples and blasting with Al_2_O_3_ or ZrO_2_ was considerably influenced by HF etching. In the case of the specimens with Al_2_O_3_ blasting + HF etching, the surface roughness increased from (Ra = 1.337, Rz = 8.594 µm) to (Ra = 2.043, Rz = 11.742 µm). For the specimens of ZrO_2_ blasting + HF etching, the surface roughness increased from (Ra = 0.726, Rz = 5.533 µm) to (Ra = 1.336, Rz = 10.353 µm).

## Figures and Tables

**Table 1:** Chemical analysis of Ti64 powder and ASTM specification.

(Mass%)		Al	V	Fe	O	N	C	H	Ti
Ti6Al4V powder		6.11	4.02	0.17	0.090	0.023	0.01	0.003	Bal
ASTM B348 Gr.23 [12]	Max	6.75	4.50	0.40	0.20	0.05	0.08	0.015	Bal
	Min	5.50	3.50	-	-	-	-	-	-

**Table 2:** The Ra and Rz values of the surface of the Ti6Al4V alloys manufactured by 3D printing, casting, and wrought after surface modifications.

Production Technology	Surface Treatment	Surface Roughness, Ra (μm)				Roughness Height, Rz (μm)			
		Specimen Number			Average	Specimen Number			Average
		1	2	3		1	2	3	
Casting	Polishing	0.479	0.531	0.499	0.503	3.397	3.506	3.816	3.573
Casting	Etching	0.312	0.353	0.367	0.344	2.317	2.889	2.964	2.723
Casting	Al_2_O_3_ Blasting	1.208	1.140	1.360	1.236	9.329	7.482	8.265	8.359
Casting	Al_2_O_3_ Blasting + etching	1.423	1.468	1.508	1.466	11.703	8.000	8.580	9.428
Casting	ZrO_2_ Blasting	0.734	1.049	0.916	0.900	7.499	9.777	6.417	7.898
Casting	ZrO_2_ Blasting + etching	0.804	1.307	1.061	1.075	8.198	9.843	8.670	8.904
Wrought	Polishing	0.360	0.475	0.555	0.463	2.497	3.083	3.679	3.086
Wrought	Etching	0.332	0.499	0.556	0.462	2.416	3.310	3.610	3.112
Wrought	Al_2_O_3_ Blasting	0.493	0.634	0.823	0.650	3.515	4.415	6.222	4.171
Wrought	Al_2_O_3_ Blasting + etching	0.729	0.877	1.215	0.940	6.144	5.982	10.953	7.693
Wrought	ZrO_2_ Blasting	1.329	1.460	1.415	1.401	9.017	7.951	8.965	8.644
Wrought	ZrO_2_ Blasting + etching	1.519	1.636	1.738	1.631	10.001	10.462	12.397	10.953
3D printing	Polishing	0.474	0.701	0.502	0.559	4.079	4.915	3.4516	4.149
3D printing	Etching	0.755	0.995	0.579	0.776	4.787	5.974	2.923	4.561
3D printing	Al_2_O_3_ Blasting	1.328	1.239	1.444	1.377	9.091	7.507	9.183	8.594
3D printing	Al_2_O_3_ Blasting + etching	2.623	1.763	1.743	2.043	12.625	11.252	11.349	11.742
3D printing	ZrO_2_ Blasting	0.715	0.677	0.786	0.726	6.103	5.024	5.473	5.533
3D printing	ZrO_2_ Blasting + etching	1.549	1.557	0.903	1.336	11.618	12.693	6.748	10.353

## Data Availability

The data used for the research are available upon request.
